# *DMD* transcripts in CRL-2061 rhabdomyosarcoma cells show high levels of intron retention by intron-specific PCR amplification

**DOI:** 10.1186/s12935-017-0428-4

**Published:** 2017-05-23

**Authors:** Emma Tabe Eko Niba, Ryo Yamanaka, Abdul Qawee Mahyoob Rani, Hiroyuki Awano, Masaaki Matsumoto, Hisahide Nishio, Masafumi Matsuo

**Affiliations:** 1 Department of Physical Therapy, Faculty of Rehabilitation, Kobe Gakuin University, 518 Arise, Ikawadani, Nishi, Kobe, 6512180 Japan; 2 Department of Pediatrics, Kobe University Graduate School of Medicine, Chuo, Kobe, 6500017 Japan; 3 Department of Community Medicine and Social Healthcare Sciences, Kobe University Graduate School of Medicine, Chuo, Kobe, 6500017 Japan

**Keywords:** *DMD*, Intron retention, Rhabdomyosarcoma, Tumor suppressor

## Abstract

**Background:**

The *DMD* gene encoding dystrophin is mutated in Duchenne muscular dystrophy, a fatal progressive muscle wasting disease. *DMD* has also been shown to act as a tumor suppressor gene. Rhabdomyosarcoma (RMS) is a mesodermal sarcoma that shares characteristics of skeletal muscle precursors. Products of the *DMD* gene in RMS have not yet been fully clarified. Here, *DMD* products were analyzed in CRL-2061 cells established from alveolar RMS.

**Methods:**

The 14-kb long *DMD* cDNA was PCR amplified as 20 separated fragments, as were nine short intron regions. Dystrophin was analyzed by Western blotting using an antibody against the C-terminal region of dystrophin.

**Results:**

Sixteen of the 20 *DMD* cDNA fragments could be amplified from CRL-2061 cells as muscle cDNA. Three fragments included aberrant gene products, including one in which exon 71 was omitted and one each with retention of introns 40 and 58. In one fragment, extending from exon 70 to 79, no normally spliced product was obtained. Rather, six alternatively spliced products were identified, including a new product deleting exon 73, with the most abundant product showing deletion of exon 78. Although dystrophin expression was expected in CRL-2061 cells, western blotting of cell lysates showed no evidence of dystrophin, suggesting that translation of full-length *DMD* mRNA was inhibited by intron retention that generated a premature stop codon. Intron specific PCR amplification of nine short introns, showed retention of introns 40, 58, and 70, which constituted about 60, 25 and 9%, respectively, of the total PCR amplified products. The most abundant DMD transcript contained two abnormalities, intron 40 retention and exon 78 skipping.

**Conclusions:**

Intron-specific PCR amplification showed that *DMD* transcripts contained high levels of introns 40, 58 and 70. Retention of these introns may have been responsible for the lack of dystrophin expression by CRL-2061 cells, thereby abolishing the tumor suppressor activity of dystrophin.

## Background

Rhabdomyosarcoma (RMS) is the most common soft-tissue sarcoma of childhood and adolescence [1]. Because RMS cells possesses characteristics of skeletal muscle precursor cells [1], expression of muscle protein has been used as a marker of RMS [2]. RMS can be divided into two broad histopathologic subtypes: embryonal RMS (ERMS) and alveolar RMS (ARMS) [3, 4]. ERMS is the most common variant, with more favorable outcomes than other variants; whereas ARMS is the second most common variant, being associated with a poor prognosis [5]. The presence of a *PAX*–*FOXO* fusion gene is associated with the poorer prognosis [6]. Further molecular characterization of ARMS may therefore reveal new therapeutic targets [1].

The *DMD* gene is the largest human gene, spanning more than 2.4 Mb on chromosome X and composed of 79 exons. This gene encodes a 14-kb transcript, which produce dystrophin, a 427 kDa protein [7]. Dystrophin deficiency caused by mutations in the *DMD* gene is a fundamental defect in Duchenne muscular dystrophy (DMD), one of the most common inherited muscular diseases. DMD is characterized by muscle weakness, leading to fatal progressive muscle wasting, as well as other complications [8, 9]. In particular, two DMD patients have been reported to show complications of RMS [10, 11].

The involvement of the *DMD* gene in tumorigenesis has indicated that dystrophin may act as a tumor suppressor. Somatic deletions of *DMD* exons have been identified in cancers with myogenic programs, enhancing their metastatic potential [12]. In addition, decreased dystrophin expression was found associated with lower survival rate in patients with gastrointestinal cancers [13]. Furthermore, *DMD* mutations were found associated with significantly poorer survival among patients with non-myogenic cancer [14], and deletions in exons of the *DMD* gene were reported in four patients with ERMS [12]. Although dystrophin expression has been assessed in RMS as a marker of skeletal muscle differentiation, dystrophin has not been fully studied in patients with ARMS [2].

Intron retention may result from genomic mutations that destroy conserved splicing regulatory sequences [15]. Alternatively, intron retention may result from mis-splicing, as shown by the incorporation of a premature stop codon into the mRNA, which is detected at extremely low levels [16]. Recently, ultra-deep high throughput RNA sequencing has revealed numerous transcripts with retained introns [17, 18]. Intron retention may play a role in regulating gene expression or in generating new mRNA isoforms [17, 19]. In particular, intron retention has been shown to be involved in tumorigenesis, by abolishing the expression of tumor suppressor genes in various cancers [18]. Introns retained by *DMD* mRNA have been identified as either pseudo-exons [20, 21] or as an unspliced intron [22], but the involvement of these DMD introns in the disease process remains to be clarified.

The CRL-2061™ (SJ-RH30) cell line was established from the bone marrow of a boy with ARMS [23] and was shown to possess the *PAX3*–*FOXO1* fusion gene [24]. This cell line has been widely used, not only in studies on tumorigenesis and cancer therapy [25, 26], but as a skeletal muscle surrogate [27]. However, the dystrophin gene expressed by these cells has not been fully characterized. This study shows that CRL-2061 cells did not express dystrophin, despite the presence of *DMD* cDNA. High levels of retained introns were detected in CRL-2061 *DMD* cDNA, suggesting that intron retention abolished dystrophin production and disrupted the tumor suppressor gene *DMD* in CRL-2061 cells.

## Methods

### Cell line

The CRL-2061™ (SJ-RH30) cell line was purchased from the American Type Culture Collection (ATCC; Manassas, VA, USA) within the last 2 years. These cells were cultured in RPMI medium (Gibco Life Technologies, Grand Island, NY, USA) supplemented with 10% fetal bovine serum (FBS; Gibco Life Technologies), and 1% antibiotic–antimycotic solution (Gibco Life Technologies) at 37 °C in a 5% CO_2_ humidified incubator. Cultured cells were rinsed twice with phosphate buffered saline (PBS; Sigma-Aldrich Co., St. Louis, MO, USA) and collected in lysis/binding buffer of High Pure RNA isolation kits (Roche Diagnostics, Basel, Switzerland).

### Transcript analysis

RNA was extracted from cells according to the manufacturer’s instructions (Roche Diagnostics). Human total skeletal muscle RNA was obtained from a human total RNA Master Panel II (Clontech Laboratories, Inc., Mountain View, CA, USA). cDNA was synthesized from 0.5 µg of each total RNA using random primers as described [28]. The *DMD*-transcript was PCR amplified as 20 separate fragments [21, 29]. Promoter specific transcripts and 9 short *DMD* intron regions were also PCR amplified [22], as were transcripts of the *PAX3*–*FOXO1* fusion gene [30] and the glyceraldehyde dehydrogenase (GAPDH) gene [31].

PCR amplification was performed in a total volume of 10 µl, containing 1 µl of cDNA, 1 µl of 10× ExTaq buffer (Takara Bio, Inc., Shiga, Japan), 0.25 U of ExTaq DNA polymerase (Takara Bio, Inc.), 500 nM of each primer, and 250 µM dNTPs (Takara Bio, Inc.). The amplification protocol consisted of an initial denaturation at 94 °C for 5 min, followed by 30 cycles of denaturation at 94 °C for 0.5 min, annealing at 59 °C for 0.5 min, and extension at 72 °C for 1 min on a Mastercycler Gradient PCR machine (Eppendorf, Hamburg, Germany). Amplified PCR products were electrophoresed and semiquantitated using a DNA 7500 LabChip kit on an Agilent 2100 Bioanalyzer (Agilent Technologies, Santa Clara, CA, USA). Each sample was assayed in triplicate, and the peak height of each band was quantified and averaged.

### Analysis of genomic DNA

Genomic DNA was extracted from CRL-2061 cells as described [32]. The presence of all 79 exons of the *DMD* gene in the genome was analyzed by a multiplex ligation-dependent probe amplification assay, performed by the LSI Medience Co. (Tokyo, Japan) using MLPA kit P034/035 DMD/Becker (MRC-Holland Co).

### DNA sequencing

PCR-amplified products visualized by agarose gel electrophoresis were excised from the gel with a sharp razor blade, pooled, and purified using QIAGEN gel extraction kits (QIAGEN, Inc., Hilden, Germany). Purified products were subcloned into the pT7 blue T vector (Novagen, Inc., San Diego, CA, USA) and sequenced by Greiner Japan Co. Ltd (Tokyo Japan).

### Western blotting

Dystrophin was analyzed by Western blotting. Protein samples were extracted using Cell Lysis Buffer (Cell Signaling Technology Inc., Danvers, MA, USA) containing protease inhibitor. Control muscle lysates were obtained from a normal immortalized cell line [33]. The protein extracts were mixed equal volumes of Laemmli Sample Buffer (Bio-Rad Laboratories, Inc., Hercules, CA, USA) and boiled for 3 min. Proteins were resolved on Mini-PROTEAN^®^ TGX Precast Gels 10% (Bio-Rad Laboratories, Inc.), using HiMark™ Pre-Stained Protein Ladder (Thermo Fisher Scientific, Inc.) as a protein size marker, and electro-transferred onto PVDF membranes (Bio-Rad, Laboratories, Inc.). The membranes were blocked with 2% ECL Prime Blocking Reagent (GE Healthcare) and incubated overnight with a 1:1000 dilution of a rabbit monoclonal antibody against a synthetic peptide corresponding to amino acids 3661–3677 of the C-terminal domain of human dystrophin (ab15277; Abcam, Cambridge, UK), followed by incubation with anti-rabbit IgG secondary antibody (GE Healthcare). As a loading control, membranes were incubated with a 1:2000 dilution of a mouse monoclonal antibody against actin (C4, Santa Cruz Biotechnology, Inc., Santa Cruz, CA, USA), followed by incubation with anti-mouse IgG secondary antibody (GE Healthcare). Immunoreactive bands were detected with ECL Select Western Blotting Detection Reagent (GE Healthcare).

## Results

**Fig. 1:**
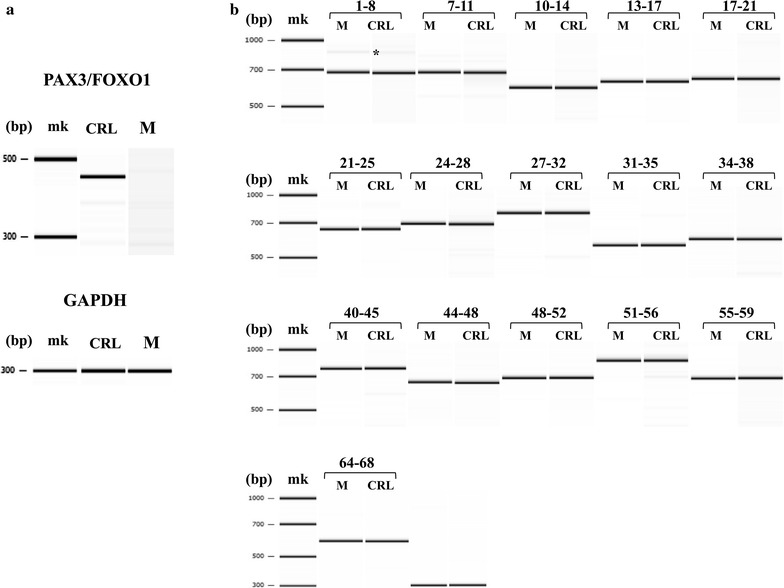
Analyses of transcripts in CRL-2061 cells. *MK* molecular size markers. **a** PCR amplification of the *PAX3/FOXO1* fusion gene transcript. The junction of the fusion gene transcript was PCR amplified. The expected size product was obtained from CRL-2061 cells (CRL), but not from muscle (M). As a control, GAPDH was also amplified (GAPDH). **b** PCR amplification of *DMD* cDNA from CRL-2061 cells. *DMD* cDNA was PCR amplified as 20 separate fragments. Of the 16 products shown, 15 were of the same sizes in CRL-2061 (CRL) cells and muscle (M). Two products were obtained after amplifying exons *1*–*8* from both the CRL and M samples. The major band consisted of exons *1*–*8*, and the minor, but larger band showed insertion of exon 1a between exons 1 and 2(*). The *numbers* above the electrophoregrams indicate the 5′ and 3′ exons of each amplified fragment

To assess the quality of CRL-2061 cDNA preparations, *PAX3*–*FOXO1* transcripts were PCR amplified, yielding a single product of expected size (Fig. 1a). PCR amplification of *GAPDH* transcripts also yielded a single product of expected size, confirming that these cDNA preparations did not contain any genomic DNA.

To assess possible genetic alterations in the *DMD* gene transcripts of CRL-2061 cells, it was first necessary to determine whether genome *DMD* was intact. Multiplex ligation-dependent probe amplification assays showed that all 79 *DMD* exons were present in genomic DNA of CRL-2061 cells (data not shown), indicating that the genomic structure of the *DMD* gene is normal in this ARMS cell line.

To analyze *DMD* transcripts in CRL-2061 cells, full-length *DMD* cDNA was RT-PCR amplified as 20 partially overlapping fragments. 16 of the 20 fragments were successfully amplified in CRL-2061 and muscle cDNA preparations (Fig. 1b). While 15 fragments in both cDNA preparations were amplified as single products of expected size, the 5′-most sequence, extending from exon 1 to exon 8, was amplified as two products, a major and a minor product (Fig. 1b). The major product was normally spliced and consisted of exons 1–8, whereas the minor product contained a cryptic exon 1a between exons 1 and 2, as reported in lymphocytes [20]. Exon 1a encodes a premature stop codon, and a cDNA containing this exon cannot produce dystrophin.

**Fig. 2:**
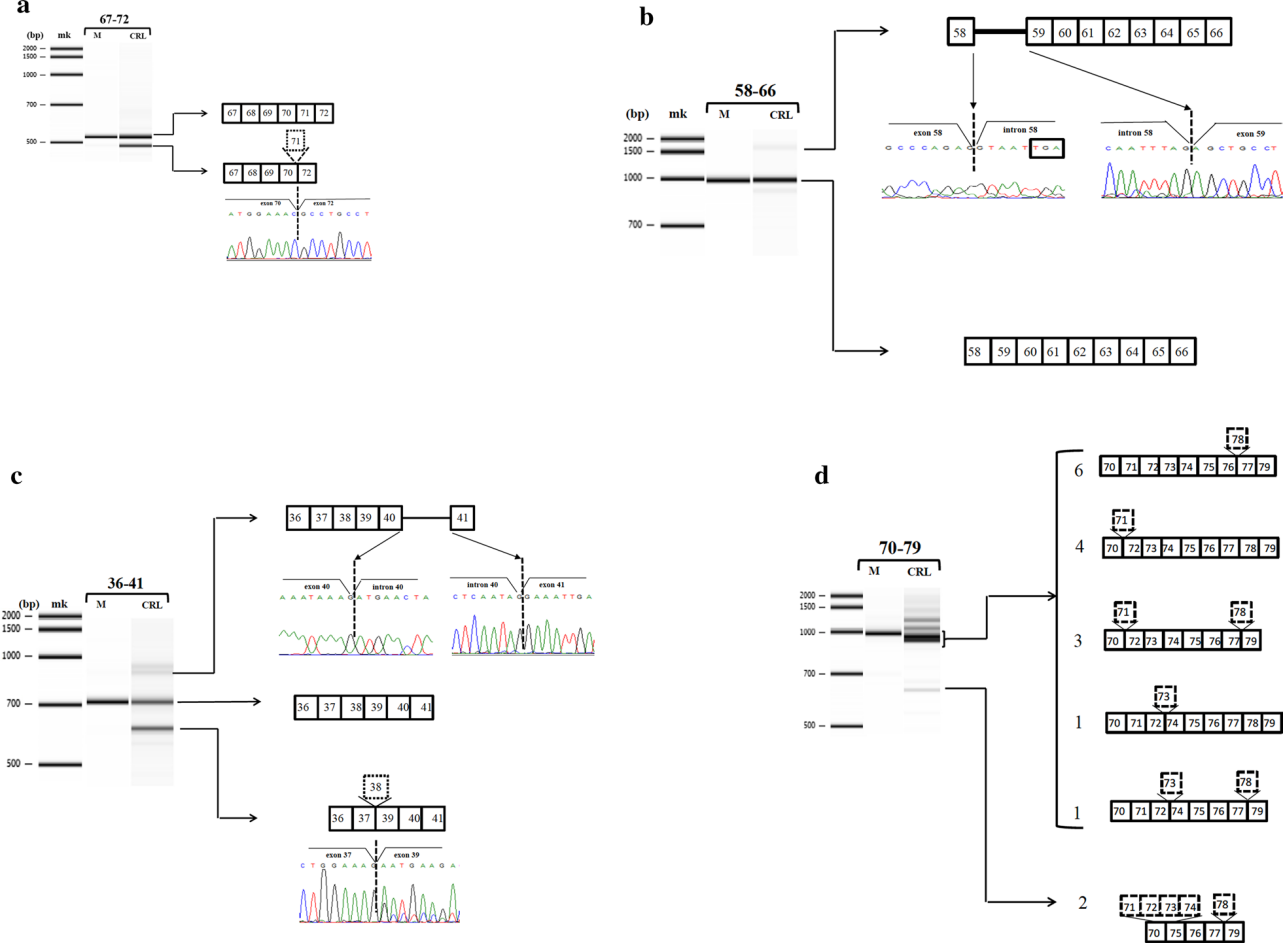
Electropherograms of PCR-amplified products of parts of the *DMD* transcript. *MK* molecular size marker. **a** Exons 67–72. A single amplified product was obtained from muscle (M), whereas two products obtained from CRL-2061 cells (CRL). The upper band in the latter was the normally spliced product, whereas the smaller sized product lacked exon 71 completely. The exon structure of the two products is shown schematically on the *right*. *Boxes* and *numbers in the boxes* represent exons and exon numbers, respectively. Partial nucleotide sequences at the junctions between exons 70 and 72 are shown *under the boxes*. **b** Exons 58–66. A single amplified product was obtained from muscle (M), whereas three products were obtained from CRL-2061 cells (CRL). The major band was the normally spliced product. Sequencing showed that the smallest-sized bane was a non-specific product, whereas the largest band contained full-length intron 58 between exons 58 and 59. The exon structure of the two products is shown schematically on the right. *Boxes* and *numbers in the boxes* represent exons and exon numbers, respectively. *Bar* indicates intron 58. Partial nucleotide sequences at the junctions between exon 58 and intron 58, and between intron 58 and exon 59 are shown *under the boxes*. The third codon in intron 58 is a TGA stop codon (*box*). **c** Exons 36–41. A single amplified product was obtained from muscle (M), whereas four bands, two dense and 2 weak, were obtained from CRL-2061 cells (CRL). The upper dense band was the normally spliced product and the lower dense band lacked exon 38. Sequencing showed that the largest sized product was non-specific, whereas the other large product contained exon 41e between exons 40 and 41. The exon structure of the two products is shown schematically on the *right*. *Boxes* and *numbers in the boxes* represent exons and exon numbers, respectively. *Bar* indicates intron 40. Partial nucleotide sequences under the boxes show the junctions between exons 38 and 39, exon 40 and intron 40, and intron 40 and exon 41. **d** Exons 70–79. A single amplified product was obtained from muscle (M), whereas several bands were obtained from CRL-2061 cells (CRL). The CRL sample lacked the band observed in M, but contained a single smaller, but broader band as its major product. Subcloning and sequencing showed that the larger sized products were non-specific. The smallest band represented an amplified product with deletions of exons 71–74 and 78. The major broad bands consisted of five products. Six clones showed deletion of exon 78, four showed deletion of exon 71, and three showed deletion of both. One clone showed deletion of exon 73 alone and one showed deletions of exons 73 and 78. The exon structures of these products are shown schematically on the *right*, with numbers to the *left* of these structures indicating the number of sequenced clones. *Boxes* and *numbers in the boxes* represent exons and exon numbers, respectively

Four sets of primers amplified multiple cDNA products in CRL-2061 cells, but single products from normal muscle. The sequence encoding exons 67–72 in CRL-2061 cDNA was visualized as two bands (Fig. 2a), with the larger band being the normally spliced product and the smaller band showing complete deletion of exon 71. Exon 71 is 39-bp long, with the cDNA containing skipped exon 71 maintaining the normal reading frame of dystrophin cDNA [34]. The fragment encoding exons 58–66 was visualized as three bands (Fig. 2b), with the middle-sized band, of highest density, being the normally spliced product. The smallest band was found to be a non-specific product, whereas the largest band showing the presence of full-length intron 58 between exons 58 and 59. Retention of intron 58 resulted in the introduction of a stop codon, being the third codon in intron 58. The fragment encoding exons 36–41 was visualized as four amplified bands, two dense and 2 weak bands (Fig. 2c). The upper dense band was the normally spliced product, consisting of exons 36–41. The lower dense band lacked exon 38; because this exon is 123-bp long, the cDNA reading frame was maintained [35]. Of the 2 weakly visualized bands, the larger was non-specific, whereas the smaller contained 568-bp of intron 40 between exons 40 and 41. This insertion introduced a premature stop codon [35]. No clone showed both exon 38 skipping and intron 40 retention, indicating that these two anomalies were mutually exclusive. These results clearly indicate that intron retention can be identified by conventional *DMD* cDNA analysis.

Notably, amplification of the fragment encoding exons 70–79 yielded multiple bands (Fig. 2d). To confirm their identity, each of these bands was subcloned and sequenced. The smallest band was found to lack exons 71–74 and exon 78. The band of highest density was smaller and broader in size than that from muscle. Seventeen clones were sequenced, yielding five separate transcripts. Six clones showed deletion of exon 78, four showed deletion of exon 71, and three showed deletion of both. One clone lacked exon 73 and one lacked exons 73 and 78. Two large size bands were found to be non-specific products. Thus, amplification of this region showed six species of transcripts, resulting from alternative splicing. Twelve of the 17 clones lacked exon 78, indicating the importance of exon 78 skipping. This enabled production of dystrophin with an elongated C-terminal end. Although skipping of exon 73 along with neighboring exons has been reported [34], this study is the first, to our knowledge, to show skipping of exon 73 alone.

**Fig. 3:**
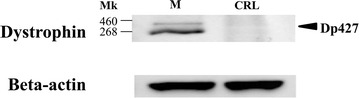
Western blotting for expression of dystrophin. Lysates of CRL-2061 cells (CRL) and human muscle (M) were subjected to Western blot analysis using an antibody against the C-terminal region of dystrophin. Dystrophin was detected in M but not in CRL (*Top*), whereas β-actin, the loading control, was positive in both (*Bottom*). Size markers are shown at *left*

These results showed that full-length *DMD* mRNA was produced in CRL-2061 cells. To extend these findings, dystrophin expression was assayed in CRL-2061 lysates by Western blotting, using an antibody against the C-terminal region of dystrophin. Although present in muscle tissue, full-length dystrophin was not detected in CRL-2061 cells (Fig. 3). Because the full-length *DMD* transcript was present in these cells, these findings indicated translational arrest of *DMD*. Although retention of introns 40 and 58 has been hypothesized to be a mechanism underlying translational arrest, conventional cDNA analyses showed that little abundance of amplified products containing retained intron 40 or 58.

**Fig. 4:**
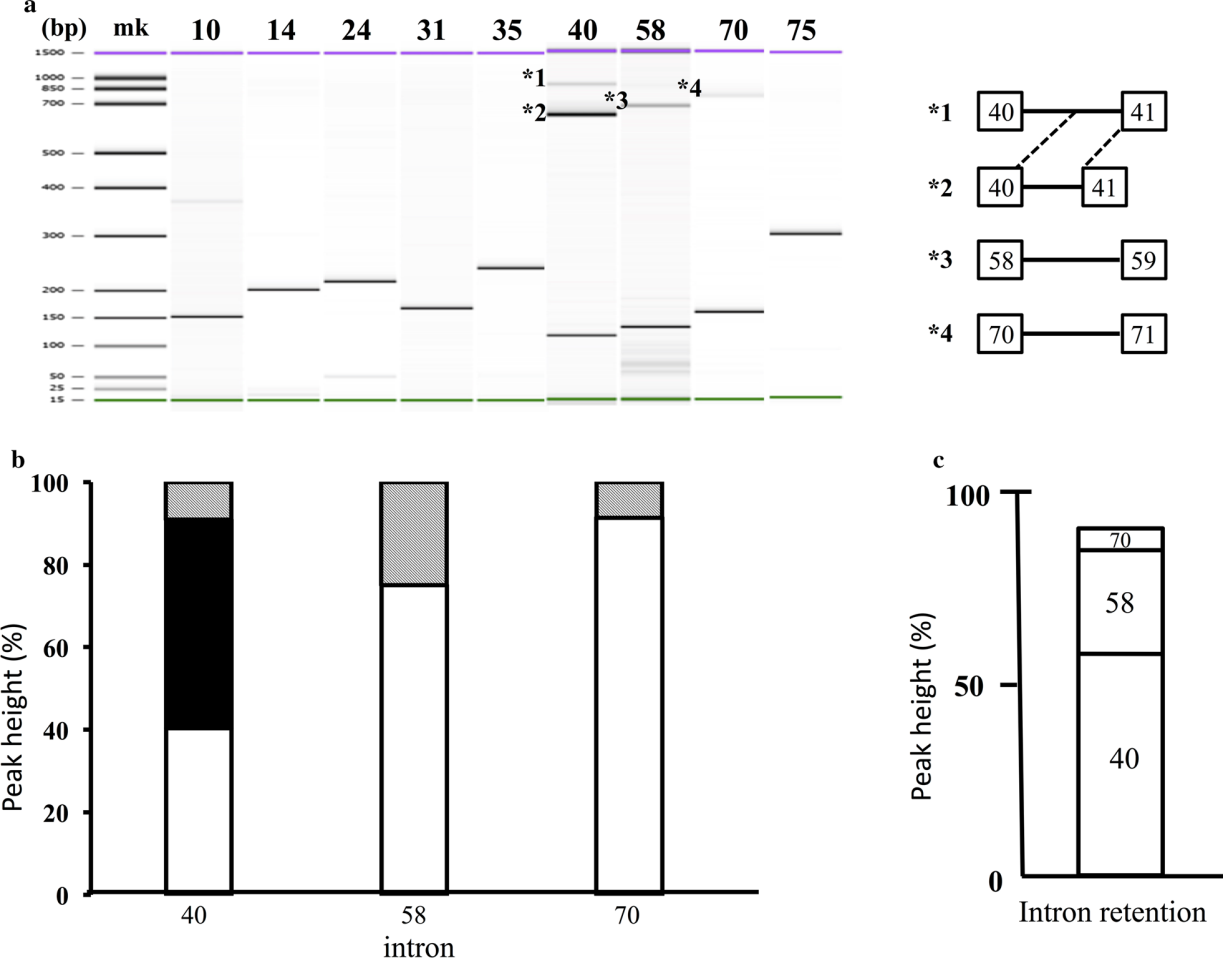
PCR amplification of 9 short intron regions in *DMD* mRNA. **a** Nine short amplified products. Electrophoregrams showing amplification of nine introns, using primers for upstream and downstream exons. Amplifications of regions covering introns 10, 14, 24, 31, 35 and 75 each yielded one band, corresponding to normally spliced products. Amplification of regions covering introns 40, 58 and 70, however, each yielded multiple bands. Amplification of intron 40 yielded three bands. The densest band included a 568-bp fragment of intron 40 (exon 41e) between exons 40 and 41 (*2); the next dense band corresponded to the normally spliced product, consisting of exons 40 and 41; and the largest band contained the full-length of intron 40 between exons 40 and 41 (*1). Amplification of intron 58 yielded three bands. The largest was non-specific; the middle band contained intron 58 between exons 58 and 59 (*3), and the smallest corresponded to the normally spliced product. Amplification of intron 70 yielded two bands, the major band being the normally spliced product, and the larger band containing intron 70 between exons 70 and 71 (*4). The structures of intron retaining products are shown schematically on the right. *Boxes* and *numbers in the boxes* represent exons and exon numbers, respectively. *Bars* indicate introns. **b** Quantification of amplified products. The peak height of each product was determined semi-quantitatively by the Bioanalyzer, and the percentage of each product relative to all amplified products (*vertical bars*) was calculated. *Clear* and *shaded boxes* represent normal and intron retained products, respectively. The *black box* represents the product retaining part of intron 40. **c** Accumulated percentage of three retained introns. The percentage of products with retained introns 40, 58 and 70 is represented by *open boxes*. Half of the product containing intron 70 lacked exon 70, reducing by half the percentage determined by intron specific PCR. The accumulated percentage was close to 90%. *Numbers in the box* indicate intron number

Intron retention in *DMD* mRNA was further investigated by amplifying nine short introns (i.e. introns 10, 14, 25, 31, 35, 40, 58, 70 and 75), using primers on neighboring exons [22]. Amplification of six of these nine introns (introns 10, 14, 24, 31, 35 and 75) yielded single products of expected size, indicating normal splicing of these introns (Fig. 4a). In contrast, amplification of two introns (introns 58 and 70) yielded two products each (Fig. 4a). The denser band resulting from amplification of intron 58 was the normally spliced product, consisting of two exons (58 and 59), whereas the larger, but less dense band consisted of full-length intron 58 inserted between exons 58 and 59. This finding was in agreement with the results of conventional cDNA analysis (Fig. 2). However, the level of intron retention was high, with 24.9% of the amplified product containing intron 58 (Fig. 4b). Similarly, the denser band resulting from amplification of intron 70 was identified as the normally spliced product, whereas the larger, but less dense band, accounting for 8.6% of amplified product, contained full-length intron 70 (Fig. 4b). The incorporation of intron 70 into *DMD* cDNA introduced a stop codon at the seventh codon of intron 70.

Amplification of intron 40 yielded three bands, with the smallest being the normally spliced product consisting of exons 40 and 41 (Fig. 4a). The middle-sized contained a 568-bp insertion, corresponding to the 3′ end of intron 40 (exon 41e), between exons 40 and 41 [33]. The largest band included the full-length intron 40 between exons 40 and 41 [22]. The relative densities of these three bands were 40.3, 50.6, and 9.1%, respectively (Fig. 4b), indicating that about 60% of the amplification products included retained introns.

These intron retentions suggested somatic nucleotide changes in these regions. Examination of all amplified products containing retained introns 40, 58, and 70 for any somatic nucleotide changes showed that all sequences at splicing donor and acceptor sites, as well as sequences of flanking exons, were wild-type. Some of these retained introns introduced premature stop codons into the dystrophin reading frame, causing translational arrest. However, these partial intron retentions could not explain the total absence of dystrophin in CRL-2061 cells. Rather, the complete absence of dystrophin would require every transcript to retain at least one intron. Post-transcriptional modification of *DMD* transcripts by intron retention at only one but not multiple sites in each transcript may result in additivity of transcripts with at least one retained intron. The accumulated percentage of transcripts with at least one retained intron retention was nearly 90% (Fig. 4c), indicating that almost all transcripts have one intron retention. Although possibly explaining the absence of dystrophin production in the cells, further studies are required to confirm this hypothesis.

## Discussion

This study showed that CRL-2061 cells, established from a patient with ARMS, lacked dystrophin, despite expressing full-length *DMD* transcripts on conventional cDNA analysis. Non-expression of dystrophin was unexpected, because CRL-2061 cells share characteristics of skeletal muscle precursors and are used experimentally as skeletal muscle surrogates [27]. Although this discrepancy may be due to a loss of muscle lineage characteristics, CRL-2061 cells showed muscle-specific promoter activation of the *DMD* gene. In contrast, about 90% of *DMD* transcripts showed retention of three introns (introns 40, 58 and 70), which may have abolished dystrophin production. These results indicated that post-transcriptional modifications, not genomic mutations, caused this genetic defect in these cells. However, intron retention may occur at two sites, not at one [18, 36].

Three studies have assessed dystrophin in cancers. One showed somatic genomic exon deletions in cancers with myogenic programs [12], whereas the other two showed that dystrophin expression was reduced in gastrointestinal cancers [13] and in other non-myogenic cancers [14]. Our results suggest that intron retention may reduce dystrophin expression. Secondary gene alterations are rare in ARMS initiated by PAX3/7–FOXO gene fusion [37]. Our results indicated that the *DMD* gene in CRL-2061 cells is structurally normal, although its transcripts are abnormal, suggesting that post-transcriptional modifications of *DMD* should be analyzed as a secondary gene alteration in cancers.

Although conventional cDNA analysis revealed intron retentions, these products constituted only a small percentage of the PCR products, making it difficult to conclude that intron retention led to the complete absence of dystrophin expression. In contrast, intron specific amplification revealed a high level of intron retention. For example, amplification of intron 40 showed that intron retention in nearly 60% of the PCR products. The discrepant results of these two assays were likely due to differences in the efficiency of PCR amplification. Moreover, amplified products were separated using a DNA7500 LabChip on the Bioanalyzer, a highly efficient apparatus able to clearly separate extra-large products.

Intron retention may result in a disease phenotype by altering the mRNA reading frame, or it may result in a mis-spliced product. Intron retention may be the second switch, after transcription, that regulates protein expression [19]. The *DMD* gene is characterized by very large introns, with the retention of part of intron 1 by mRNA first reported to be a cryptic exon [20]. Subsequently, 14 cryptic exons were identified [21], but their role has not been determined. Intron retention is a widespread mechanism for inactivation of tumor-suppressor genes [18, 38]. Dystrophin has been shown to act as a tumor suppressor [12–14], making intron retention in the *DMD* a likely mechanism of tumor suppressor inactivation.

Two types of intron 40 retention were observed in CRL-2061 cells, retention of full-length and part of intron 40. Similarly, *DMD* transcripts of SH-SY5Y neuroblastoma cells showed retention of part of intron 40 [35], suggesting that alternate splicing of this intron is specific to cancer cells. Tumor cells show many alternative splicing events associated with tumor progression and metastasis, and the deregulation of splicing factors [39]. Intron retention has been observed in cancers even in the absence of mutational insults to the splicing machinery [38]. In contrast, intron retention has also been observed to result from single nucleotide variants near splice junctions [18]. The mechanisms regulating *DMD* intron 40 retention should be further clarified in other tumor-derived cells.

To our knowledge, this study is the first to show skipping of *DMD* exon 73 alone by conventional cDNA analysis. Previously, skipping of exon 73 alone in skeletal muscle was detected only by targeted RNA-Seq profiling [40]. Therefore, a high level of exon 73 skipping seemed unique to CRL-2061 cells. Skipping of exon 73 may produce dystrophin lacking an α-syntrophin binding site.

Skipping of *DMD* exon 78 was shown to be another major splicing product in CRL-2061 cells. Skipping of this exon has been reported in transcripts obtained from in skeletal muscle [34] and has been classified as fetal type [41]. Because RMS possesses the characteristics of skeletal muscle precursors [1], fetal type splicing in CRL-2061 cells was not unexpected.

Rhabdomyosarcoma may be diagnosed by the immunohistochemical or molecular detection of a myogenic regulatory factor, such as MyoD or myogenin [42]. In contrast, the expression of contractile proteins, such as myosin, is indicative of differentiated tumor phenotypes [43]. Dystrophin is regarded as an immunohistologic marker of RMS [44]. Using an antibody recognizing the mid-rod domain of dystrophin, eight of nine RMSs were found positive for dystrophin [2], whereas one showed lack of dystrophin expression. Dystrophin expression patterns can therefore differentiate between two types of RMS.

**Fig. 5:**
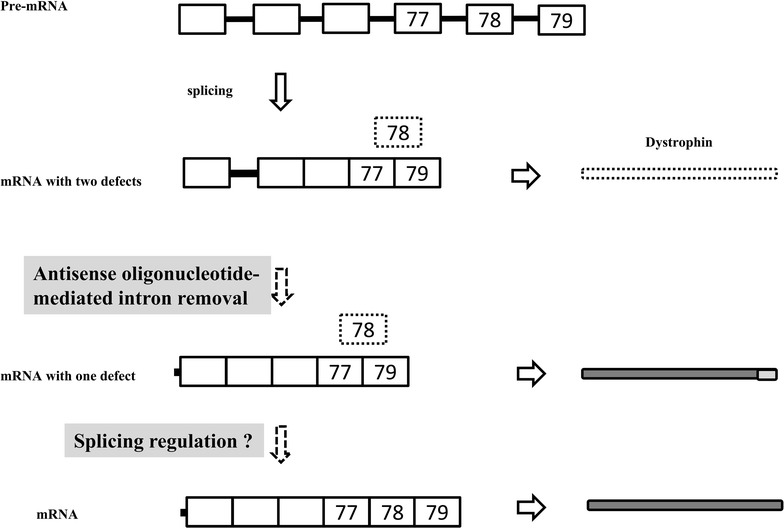
Schematic illustration of two methods of restoring dystrophin expression in CRL-2061 cells. A schematic representation of the *DMD* transcript shows the three last exons and three representative upstream exons (pre-mRNA). Splicing of the pre-mRNA produces mRNA with intron retention (*thick bar*) and exon 78 skipping (*box at the top* of the exon structure). This mRNA, with two defects, cannot produce dystrophin. Intron retention may be abolished by antisense oligonucleotide-mediated intron removal. However, this protein product differs at its C-terminal from dystrophin (*grey box*; mRNA with one defect). Splicing of exon 78 may result in the production of complete dystrophin mRNA. *Boxes* and *lines* represent exons and introns, respectively. The *number in each box* indicates exon number. Exon boxes are not drawn to scale

Dystrophin deficient RMS may be treated by induction of dystrophin expression. For example, re-expression of internally deleted mini-dystrophin has been shown to suppress the malignant phenotype of ERMS cells with a *DMD* gene harboring an exon deletion [12]. Two strategies may activate dystrophin expression in CRL-2061 cells, which have a normal *DMD* gene. Because the *DMD* transcript in CRL-2061 cells was shown to have two defects, intron retention and exon 78 skipping (Fig. 5), dystrophin production may be restored by removing the retained intron from the transcript. Dystrophin-deficient DMD patients have been treated with antisense oligonucleotides that modulate splicing to skip exons with antisense oligonucleotides [45, 46], with one of these oligonucleotides recently approved by the US Food and Drug Administration [47]. Therefore, treatment with antisense oligonucleotide may induce the removal of retained introns. Functional damage to dystrophin resulting from exon 78 skipping may be overcome by incorporating exon 78 into *DMD* transcripts, producing mRNAs containing the complete C-terminal end of dystrophin. This may be accomplished by a splicing regulatory mechanism, as suggested in the treatment of myotonic dystrophy [41].

## Conclusions

Dystrophin was not identified in CRL-2061 cells, despite the presence of full-length *DMD* cDNA. These transcripts showed high levels of retention of introns 40, 58 and 70, as well as exon 78 skipping. These findings suggest that intron retention may abolish dystrophin expression and promote tumorigenesis.

## Abbreviation

DMD: Duchenne muscular dystrophy

## References

[1] O’Brien D, Jacob AG, Qualman SJ, Chandler DS (2012). Advances in pediatric rhabdomyosarcoma characterization and disease model development. Histol Histopathol.

[2] Pinto A, Paslawski D, Sarnat HB, Parham DM (1993). Immunohistochemical evaluation of dystrophin expression in small round cell tumors of childhood. Mod Pathol.

[3] Newton WA, Soule EH, Hamoudi AB, Reiman HM, Shimada H, Beltangady M (1988). Histopathology of childhood sarcomas, intergroup rhabdomyosarcoma studies I and II: clinicopathologic correlation. J Clin Oncol.

[4] Jo VY, Doyle LA (2016). Refinements in sarcoma classification in the current 2013 World Health Organization classification of tumours of soft tissue and bone. Surg Oncol Clin N Am.

[5] Turner JH, Richmon JD (2011). Head and neck rhabdomyosarcoma: a critical analysis of population-based incidence and survival data. Otolaryngol Head Neck Surg.

[6] Sorensen PH, Lynch JC, Qualman SJ, Tirabosco R, Lim JF, Maurer HM (2002). PAX3–FKHR and PAX7–FKHR gene fusions are prognostic indicators in alveolar rhabdomyosarcoma: a report from the children’s oncology group. J Clin Oncol.

[7] Muntoni F, Torelli S, Ferlini A (2003). Dystrophin and mutations: one gene, several proteins, multiple phenotypes. Lancet Neurol..

[8] Banihani R, Smile S, Yoon G, Dupuis A, Mosleh M, Snider A (2015). Cognitive and neurobehavioral profile in boys with Duchenne muscular dystrophy. J Child Neurol.

[9] Kamdar F, Garry DJ (2016). Dystrophin-deficient cardiomyopathy. J Am Coll Cardiol.

[10] Rossbach HC, Lacson A, Grana NH, Barbosa JL (1999). Duchenne muscular dystrophy and concomitant metastatic alveolar rhabdomyosarcoma. J Pediatr Hematol Oncol.

[11] Jakab Z, Szegedi I, Balogh E, Kiss C, Olah E (2002). Duchenne muscular dystrophy-rhabdomyosarcoma, ichthyosis vulgaris/acute monoblastic leukemia: association of rare genetic disorders and childhood malignant diseases. Med Pediatr Oncol.

[12] Wang Y, Marino-Enriquez A, Bennett RR, Zhu M, Shen Y, Eilers G (2014). Dystrophin is a tumor suppressor in human cancers with myogenic programs. Nat Genet.

[13] Stephens NA, Skipworth RJ, Gallagher IJ, Greig CA, Guttridge DC, Ross JA (2015). Evaluating potential biomarkers of cachexia and survival in skeletal muscle of upper gastrointestinal cancer patients. J Cachexia Sarcopenia Muscle..

[14] Luce LN, Abbate M, Cotignola J, Giliberto F (2017). Non-myogenic tumors display altered expression of dystrophin (DMD) and a high frequency of genetic alterations. Oncotarget..

[15] Baralle D, Baralle M (2005). Splicing in action: assessing disease causing sequence changes. J Med Genet.

[16] Roy SW, Irimia M (2008). Intron mis-splicing: no alternative?. Genome Biol.

[17] Wong JJ, Au AY, Ritchie W, Rasko JE (2016). Intron retention in mRNA: no longer nonsense: known and putative roles of intron retention in normal and disease biology. BioEssays.

[18] Jung H, Lee D, Lee J, Park D, Kim YJ, Park WY (2015). Intron retention is a widespread mechanism of tumor-suppressor inactivation. Nat Genet.

[19] Ge Y, Porse BT (2014). The functional consequences of intron retention: alternative splicing coupled to NMD as a regulator of gene expression. BioEssays.

[20] Roberts RG, Bentley DR, Bobrow M (1993). Infidelity in the structure of ectopic transcripts: a novel exon in lymphocyte dystrophin transcripts. Hum Mutat.

[21] Zhang Z, Habara Y, Nishiyama A, Oyazato Y, Yagi M, Takeshima Y (2007). Identification of seven novel cryptic exons embedded in the dystrophin gene and characterization of 14 cryptic dystrophin exons. J Hum Genet.

[22] Nishida A, Minegishi M, Takeuchi A, Niba ET, Awano H, Lee T (2015). Tissue- and case-specific retention of intron 40 in mature *dystrophin* mRNA. J Hum Genet.

[23] Merlino G, Helman LJ (1999). Rhabdomyosarcoma—working out the pathways. Oncogene.

[24] Galili N, Davis RJ, Fredericks WJ, Mukhopadhyay S, Rauscher FJ, Emanuel BS (1993). Fusion of a fork head domain gene to PAX3 in the solid tumour alveolar rhabdomyosarcoma. Nat Genet.

[25] Kashima K, Watanabe M, Sato Y, Hata J, Ishii N, Aoki Y (2014). Inhibition of metastasis of rhabdomyosarcoma by a novel neutralizing antibody to CXC chemokine receptor-4. Cancer Sci.

[26] Waters AM, Stafman LL, Garner EF, Mruthyunjayappa S, Stewart JE, Mroczek-Musulman E (2016). Targeting focal adhesion kinase suppresses the malignant phenotype in rhabdomyosarcoma cells. Transl Oncol..

[27] Gitterman DP, Wilson J, Randall AD (2005). Functional properties and pharmacological inhibition of ASIC channels in the human SJ-RH30 skeletal muscle cell line. J Physiol.

[28] Matsuo M, Masumura T, Nishio H, Nakajima T, Kitoh Y, Takumi T (1991). Exon skipping during splicing of dystrophin mRNA precursor due to an intraexon deletion in the dystrophin gene of Duchenne muscular dystrophy kobe. J Clin Invest..

[29] Roberts RG, Barby TF, Manners E, Bobrow M, Bentley DR (1991). Direct detection of dystrophin gene rearrangements by analysis of dystrophin mRNA in peripheral blood lymphocytes. Am J Hum Genet.

[30] Missiaglia E, Williamson D, Chisholm J, Wirapati P, Pierron G (2012). PAX3/FOXO1 fusion gene status is the key prognostic molecular marker in rhabdomyosarcoma and significantly improves current risk stratification. J Clin Oncol.

[31] Tran VK, Zhang Z, Yagi M, Nishiyama A, Habara Y, Takeshima Y (2005). A novel cryptic exon identified in the 3′ region of intron 2 of the human dystrophin gene. J Hum Genet.

[32] Nishida A, Kataoka N, Takeshima Y, Yagi M, Awano H, Ota M (2011). Chemical treatment enhances skipping of a mutated exon in the dystrophin gene. Nat Commun..

[33] Nishida A, Yasuno S, Takeuchi A, Awano H, Lee T, Niba ET (2016). HEK293 cells express dystrophin Dp71 with nucleus-specific localization of Dp71ab. Histochem Cell Biol.

[34] Feener CA, Koenig M, Kunkel LM (1989). Alternative splicing of human dystrophin mRNA generates isoforms at the carboxy terminus. Nature.

[35] Nishida A, Minegishi M, Takeuchi A, Awano H, Niba ET, Matsuo M (2015). Neuronal SH-SY5Y cells use the C-dystrophin promoter coupled with exon 78 skipping and display multiple patterns of alternative splicing including two intronic insertion events. Hum Genet.

[36] Gazzoli I, Pulyakhina I, Verwey NE, Ariyurek Y, Laros JF, ‘t Hoen PA (2016). Non-sequential and multi-step splicing of the dystrophin transcript. RNA Biol.

[37] Chen X, Pappo A, Dyer MA (2015). Pediatric solid tumor genomics and developmental pliancy. Oncogene.

[38] Dvinge H, Bradley RK (2015). Widespread intron retention diversifies most cancer transcriptomes. Genome Med..

[39] Oltean S, Bates DO (2014). Hallmarks of alternative splicing in cancer. Oncogene.

[40] Bouge AL, Murauer E, Beyne E, Miro J, Varilh J, Taulan M (2017). Targeted RNA-Seq profiling of splicing pattern in the DMD gene: exons are mostly constitutively spliced in human skeletal muscle. Sci Rep..

[41] Rau F, Laine J, Ramanoudjame L, Ferry A, Arandel L, Delalande O (2015). Abnormal splicing switch of DMD’s penultimate exon compromises muscle fibre maintenance in myotonic dystrophy. Nat Commun..

[42] Zanola A, Rossi S, Faggi F, Monti E, Fanzani A (2012). Rhabdomyosarcomas: an overview on the experimental animal models. J Cell Mol Med.

[43] Morotti RA, Nicol KK, Parham DM, Teot LA, Moore J, Hayes J (2006). An immunohistochemical algorithm to facilitate diagnosis and subtyping of rhabdomyosarcoma: the children’s oncology group experience. Am J Surg Pathol.

[44] Gattenlohner S, Muller-Hermelink HK, Marx A (1998). Polymerase chain reaction-based diagnosis of rhabdomyosarcomas: comparison of fetal type acetylcholine receptor subunits and myogenin. Diagn Mol Pathol.

[45] Matsuo M (1996). Duchenne/Becker muscular dystrophy: from molecular diagnosis to gene therapy. Brain Dev..

[46] Aartsma-Rus A, Janson AA, Heemskerk JA, De Winter CL, Van Ommen GJ, Van Deutekom JC (2006). Therapeutic modulation of DMD splicing by blocking exonic splicing enhancer sites with antisense oligonucleotides. Ann NY Acad Sci.

[47] Stein CA (2016). Eteplirsen approved for Duchenne muscular dystrophy: the FDA faces a difficult choice. Mol Ther.

